# Interactive Domains in the Molecular Chaperone Human αB Crystallin Modulate Microtubule Assembly and Disassembly

**DOI:** 10.1371/journal.pone.0000498

**Published:** 2007-06-06

**Authors:** Joy G. Ghosh, Scott A. Houck, John I. Clark

**Affiliations:** 1 Department of Biological Structure, University of Washington, Seattle, Washington, United States of America; 2 Department of Ophthalmology, University of Washington, Seattle, Washington, United States of America; Vanderbilt University, United States of America

## Abstract

**Background:**

Small heat shock proteins regulate microtubule assembly during cell proliferation and in response to stress through interactions that are poorly understood.

**Methodology:**

Novel functions for five interactive sequences in the small heat shock protein and molecular chaperone, human αB crystallin, were investigated in the assembly/disassembly of microtubules and aggregation of tubulin using synthetic peptides and mutants of human αB crystallin.

**Principal Findings:**

The interactive sequence _113_FISREFHR_120_ exposed on the surface of αB crystallin decreased microtubule assembly by ∼45%. In contrast, the interactive sequences, _131_LTITSSLSSDGV_142_ and _156_ERTIPITRE_164_, corresponding to the β8 strand and the C-terminal extension respectively, which are involved in complex formation, increased microtubule assembly by ∼34–45%. The αB crystallin peptides, _113_FISREFHR_120_ and _156_ERTIPITRE_164_, inhibited microtubule disassembly by ∼26–36%, and the peptides _113_FISREFHR_120_ and _131_LTITSSLSSDGV_142_ decreased the thermal aggregation of tubulin by ∼42–44%. The _131_LTITSSLSSDGV_142_ and _156_ERTIPITRE_164_ peptides were more effective than the widely used anti-cancer drug, Paclitaxel, in modulating tubulin↔microtubule dynamics. Mutagenesis of these interactive sequences in wt human αB crystallin confirmed the effects of the αB crystallin peptides on microtubule assembly/disassembly and tubulin aggregation. The regulation of microtubule assembly by αB crystallin varied over a narrow range of concentrations. The assembly of microtubules was maximal at αB crystallin to tubulin molar ratios between 1∶4 and 2∶1, while molar ratios >2∶1 inhibited microtubule assembly.

**Conclusions and Significance:**

Interactive sequences on the surface of human αB crystallin collectively modulate microtubule assembly through a dynamic subunit exchange mechanism that depends on the concentration and ratio of αB crystallin to tubulin. These are the first experimental results in support of the functional importance of the dynamic subunit model of small heat shock proteins.

## Introduction

Molecular chaperones are endogenous molecules that participate in the normal folding, processing, organization, and degradation of cellular proteins including cytoskeletal proteins [1]–[3]. Human αB crystallin is the archetype of small heat shock proteins (sHSPs) which are low molecular weight (<43 kDa) chaperones that organize and stabilize the cytoskeletal networks of microfilament proteins including actin, the intermediate filaments desmin and glial-fibrillary acidic protein (GFAP), and the microtubule forming protein tubulin [4]–[16]. In the absence of stress, sHSPs interact directly with tubulin and microtubule associated proteins to promote microtubule assembly and under stress sHSPs protect against microtubule depolymerization [8], [17]–[24]. A recent report suggests that at high concentrations sHSPs inhibit rather than promote microtubule assembly [25]. The systematic characterization of the interactive domains is necessary to understand the functional importance of sHSPs in assembly of cytoskeletal proteins.

In this study, the importance of five human αB crystallin interactive sequences _41_STSLSPFYLRPPSFLRAP_58_ (ST), _73_DRFSVNLDVKHFS_85_ (DR), _113_FISREFHR_120_ (FI), _131_LTITSSLSSDGV_142_ (LT), and _156_ERTIPITRE_164_ (ER) in the assembly/disassembly of microtubules and the thermal aggregation of tubulin was evaluated using synthetic αB crystallin peptides and αB crystallin mutants. Previous protein pin array and mutagenesis studies identified these five interactive sequences in human αB crystallin for interactions with substrate proteins including lens crystallins, growth factors, and the filamentous proteins desmin, glial-fibrillary acidic protein, and actin [26]–[28]. The αB crystallin interactive sequences _131_LTITSSLSSDGV_142_ and _156_ERTIPITRE_164_ promote microtubule assembly and inhibit microtubule disassembly, while the interactive sequence _113_FISREFHR_120_ inhibited both microtubule assembly and disassembly. The remaining two peptides, _41_STSLSPFYLRPPSFLRAP_58_ and _73_DRFSVNLDVKHFS_85_ had little or no effect on microtubule assembly or disassembly. Microtubule assembly varied with the ratio of tubulin to αB crystallin resolving the apparent contradictions in the results of an αB crystallin effect on tubulin assembly [19], [21], [25]. Localization of the tubulin interactive sequences on the surface of αB crystallin and the dynamic subunit model for sHSP chaperone activity accounts for the observed effects of the synthetic αB crystallin peptides and the mutant αB crystallins on tubulin/microtubules.

## Materials and Methods

### Materials

Synthetic αB crystallin peptides DRFSVNLDVKHFS, STSLSPFYLRPPSFLRAP, FISREFHR, LTITSSLSSDGV, and ERTIPITRE were procured from Advanced ChemTech (Louisville, KY) and Genscript Corporation (Piscataway, NJ).

### Construction, expression, and purification of wt and mutant αB crystallins

The αB crystallin mutants were constructed using the Quick-Change site-directed mutagenesis kit as described previously [29]–[32]. The R120G mutant is a single point mutant of the _113_FISREFH**R**_120_ sequence of human αB crystallin, constructed by replacing Arg-120 with a glycine residue. The αAβ8 mutant was constructed by replacing the α crystallin core domain β8 sequence _131_LTITSSLS_138_ of human αB crystallin with the homologous β8 sequence _127_SALSCSLS_134_ of human αA crystallin. The Δ155–165 mutant was constructed by deleting residues _155_ERTIPITRE_165_ from the C-terminus extension of human αB crystallin. Wt αB crystallin, R120G, αAβ8, and Δ155–165 were expressed and purified as described previously [30]–[32].

### Microtubule assembly assays

The effect of selected αB crystallin peptides on the *in vitro* assembly of tubulin into microtubules was evaluated using the Microtubule Stabilization/Destabilization Assay kit (Cytoskeleton; Denver, CO) as described previously [33]. Bovine brain tubulin was dissolved to 200 µM in 80 mM PIPES, 2 mM MgCl_2_, 0.5 mM EGTA, 10 µM DAPI, 1 mM GTP pH 6.9. 8.5 µl of the tubulin was mixed with 40 µl of 80 mM PIPES, 2 mM MgCl_2_, 0.5 mM EGTA, 7.4 µM DAPI, 16% Glycerol, 1.1 mM GTP pH 6.9 and 4.3 µl of 2 mM peptide in 2.5% DMSO, 2 mM Paclitaxel (polymerization promoter) in 100% DMSO, 15 mM CaCl_2_ (polymerization inhibitor) in water, or 2.5% DMSO only. Microtubule assembly was monitored by measuring the fluorescence of DAPI, a molecule whose emission fluorescence at λ = 460 is enhanced 8-fold when it is incorporated into assembled microtubules [33]. Fluorescence of samples were continuously read on a Perkin Elmer Victor^3^ V fluorescence plate reader (Excitation λ = 355 nm, Emission λ = 460 nm) at 37°C for 45 minutes.

The effect of wt and three mutant αB crystallins, Δ41–58, αAβ8, and Δ155–165 on the *in vitro* assembly of tubulin into microtubules was evaluated using the Microtubule Stabilization/Destabilization Assay kit described above (Cytoskeleton; Denver, CO). Bovine brain tubulin was dissolved to 200 µM in 80 mM PIPES, 2 mM MgCl_2_, 0.5 mM EGTA, 10 µM DAPI, 1 mM GTP pH 6.9. 8.5 µl of the tubulin was mixed with 40 µl of 80 mM PIPES, 2 mM MgCl_2_, 0.5 mM EGTA, 7.4 µM DAPI, 16% Glycerol, 1.1 mM GTP pH 6.9 and 4.3 µl of 80 µM protein in 20 mM Tris-Cl, pH8.0 or Tris-Cl buffer only. Fluorescence of samples were continuously read on a Perkin Elmer Victor^3^ V fluorescence plate reader (Excitation λ = 355 nm, Emission λ = 460 nm) at 37°C for 45 minutes.

### Microtubule disassembly assays

The effect of αB crystallin peptides and mutants on the *in vitro* disassembly of microtubules was evaluated using the Microtubule Stabilization/Destabilization Assay kit described above (Cytoskeleton; Denver, CO) using methods described previously [33]. Microtubules were assembled at 37°C in the absence of αB crystallin peptides, αB crystallin proteins, and small molecules as described previously. Incubation of microtubules at 23°C results in spontaneous microtubule disassembly. To measure the effect on microtubule disassembly, 34 µM pre-formed microtubules were incubated with αB crystallin peptides (170 µM), wt and mutant αB crystallins (6.8 µM and 34 µM) at 23°C for 20 minutes. The decrease in DAPI fluorescence at λ = 460 nm was measured continuously for 20 minutes by exciting the samples at λ = 355 nm using a Perkin Elmer Victor^3^ V fluorescence plate reader.

### Tubulin aggregation assays

The effect of αB crystallin peptides and mutants on the thermal aggregation of tubulin was evaluated using ultra-violet spectroscopy. Bovine brain tubulin was dissolved to 200 µM in 80 mM PIPES, 2 mM MgCl_2_, 0.5 mM EGTA, pH 6.9. 4.25 µl of 0.08, 0.4, or 2 mM test peptide or protein was diluted into 40 µl of 80 mM PIPES, 2 mM MgCl_2_, 0.5 mM EGTA, pH 6.9. 8.5 µl of the 200 µM tubulin was added to each sample. Samples were heated at 52°C and the absorbance at λ = 340 nm was measured continuously for 60 minutes using a Pharmacia Biotech Ultrospec 3000. GTP and glycerol were not present in the samples because they induce the assembly of microtubules.

### Homology modeling

The tubulin interactive sequences _113_FISREFHR_120_, _131_LTITSSLS_138_, and _156_ERTIPITRE_164_ were mapped to a 3D structural model of human αB crystallin computed previously [26]. The human αB crystallin homology model was computed using the wheat sHSP16.9 X-ray crystal structure as described previously [26], [34], [35]. The Cα root mean square deviation between the superimposed model of human αB crystallin and the crystal structure of wheat sHSP16.9 was 3.25 Å. The model for the twenty-four subunit oligomer of human αB crystallin was computed using co-ordinates of the *Methanococcus jannaschii* sHSP16.5 twenty-four subunit crystal structure described previously [36].

## Results

**Figure 1:**
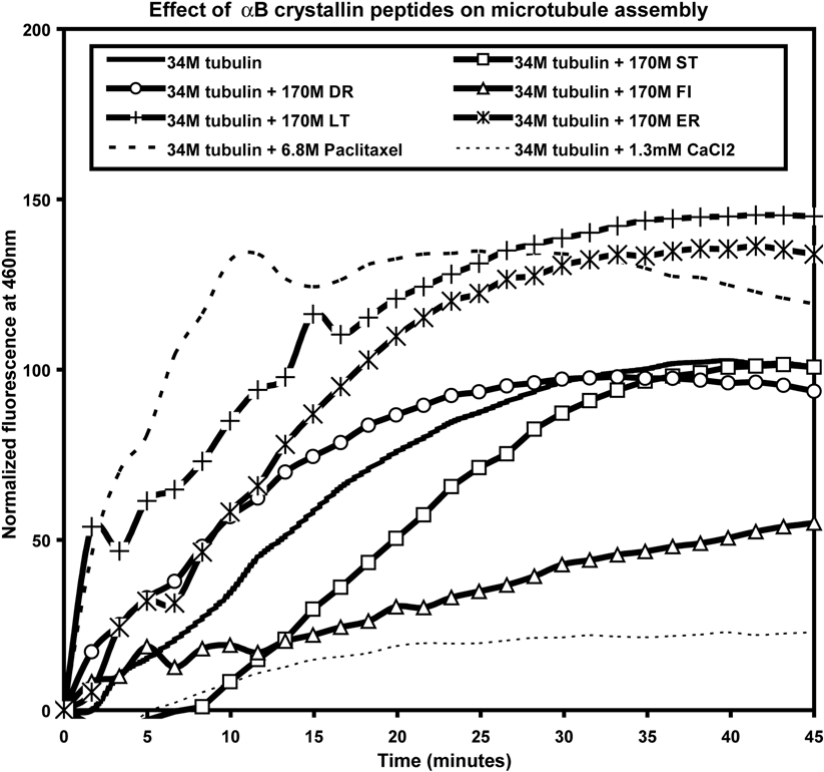
Effect of αB crystallin peptides on microtubule assembly. Samples containing tubulin and αB crystallin peptides or control molecules were excited at λ = 355 nm and the fluorescence emission of DAPI bound to assembled microtubules was recorded at λ = 460 nm. The fluorescence of the sample containing tubulin alone increased rapidly to a maximum value at 45 minutes of incubation at 37°C. The ST (N-terminus) and DR (β3) peptides had no effect on total microtubule assembly, the FI (loop) peptide inhibited microtubule assembly, while the LT (β8) and ER (C-terminus) peptides promoted microtubule assembly. The positive control, Paclitaxel, accelerated microtubule assembly, while the negative control, CaCl_2_, inhibited microtubule assembly which was consistent with previous reports [59], [60].

The effects of synthetic peptides corresponding to five human αB crystallin interactive sequences on microtubule assembly were investigated (Figure 1). When 34 µM tubulin alone was incubated at 37°C, a rapid increase in DAPI fluorescence was observed due to the preferential binding of DAPI to assembled microtubules and maximum fluorescence was observed in approximately 45 minutes. The ST peptide slowed the rate of microtubule assembly by increasing the lag phase preceding the start of microtubule assembly without an effect on the amount of microtubules formed in 45 minutes. The DR peptide accelerated microtubule assembly without an effect on the total amount of microtubules formed in 45 minutes. In contrast, the FI peptide slowed microtubule assembly and decreased the amount of microtubules formed in 45 minutes. The LT and ER peptides increased both the rate of microtubule assembly and the amount of microtubules formed in 45 minutes. The effect of the LT and ER peptides was similar to Paclitaxel, a known promoter of microtubule assembly, while the effect of the FI peptide was similar but weaker than the effect of CaCl_2_, a known inhibitor of microtubule assembly.

**Figure 2:**
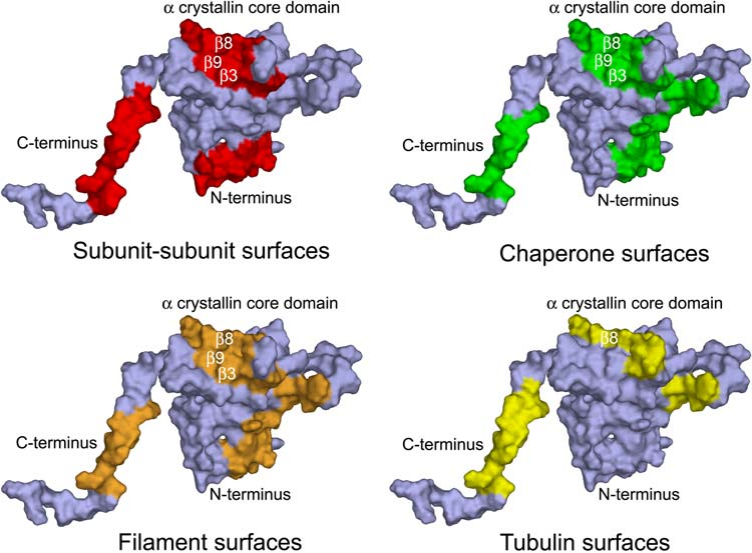
Surface locations of the interactive sequences in αB crystallin for subunit-subunit interactions, chaperone activity, and interactions with filaments and tubulin. Interactive sequences for subunit-subunit interactions, chaperone activity, and interactions with filaments and tubulin identified by *in vitro* assays, mutagenesis, and pin array analysis were mapped to the N-terminal, β3-β8-β9, and C- terminal interface regions of the human αB crystallin homology model. The ST sequence is in the N-terminal extension, the DR, LT, and FI sequences are in the β3 and β8 strands and the loop of the α crystallin core domain, and the ER sequence is in the C-terminal extension. Surfaces formed by the LT (β8) and ER (C-terminal extension containing the Ile-X-Ile motif) sequences mediated subunit-subunit interactions as well as interactions with unfolded substrate proteins, filaments, and tubulin.

**Figure 3:**
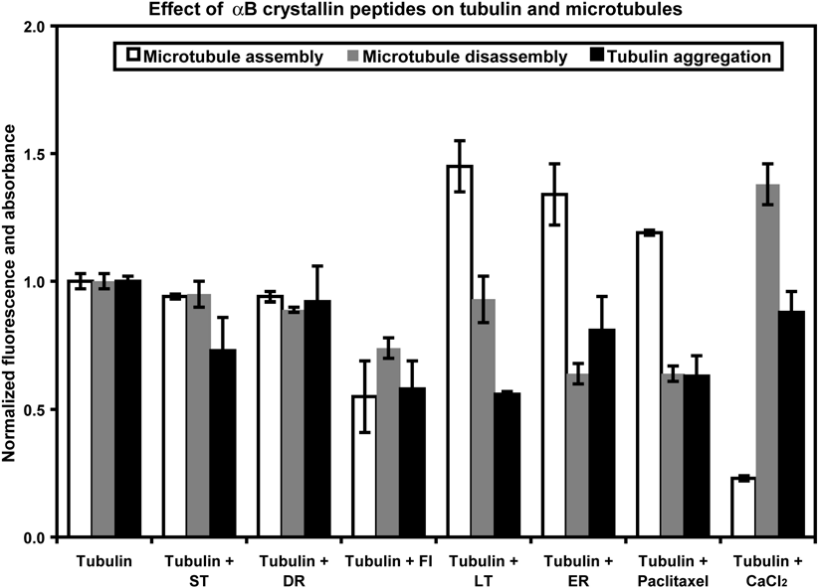
Effect of synthetic αB crystallin peptides on microtubule assembly, disassembly, and tubulin aggregation. The DAPI fluorescence of assembled microtubules, disassembled tubulin, and tubulin aggregates in the absence of αB crystallin peptides and control additives were normalized to 1.0. The FI, LT, and ER peptides had the strongest effect on microtubule assembly/disassembly and tubulin aggregation, while ST and DR peptides had little to no effect microtubule assembly/disassembly and tubulin aggregation.

Sequences in αB crystallin that altered microtubule assembly overlapped with sequences for subunit-subunit interactions chaperone activity, and filament interactions, [26], [27] (Figure 2). The overlap between αB crystallin sequences that altered microtubule assembly and αB crystallin chaperone sequences identified previously [27] suggested a functional role for αB crystallin in tubulin/microtubule stabilization. Consequently, the effects of the αB crystallin interactive sequences on microtubule disassembly and tubulin aggregation were investigated (Figure 3). Pre-formed microtubules (34 µM) were incubated in the absence and presence of αB crystallin peptides and controls at 23°C to induce disassembly of microtubules. In the absence of αB crystallin peptides and controls, microtubules alone disassembled rapidly and minimum fluorescence was recorded in approximately 20 minutes. The FI and ER peptides inhibited microtubule disassembly by ∼24% and 36% respectively similar to the microtubule-stabilizing molecule Paclitaxel, while the remaining peptides conferred little to no protection against the disassembly of microtubules.

The ability of the αB crystallin peptides to protect against the thermal aggregation of tubulin was determined by measuring the optical density (OD_340_) of 34 µM tubulin at 52°C for sixty minutes in the absence or presence of peptides and control molecules (Figure 3). In the absence of αB crystallin peptides and controls, tubulin aggregated rapidly and a maximum optical density was recorded in approximately 60 minutes. The α crystallin core domain peptides FI and LT had the strongest protective effects and decreased the aggregation of tubulin by ∼42–44%. In contrast, the N-terminal peptide ST, the α crystallin core domain peptide DR, and the C-terminal peptide, ER, had weak protective effects and the aggregation of tubulin incubated with these peptides decreased by only 8–27% relative to the control. Microtubule assembly/disassembly and thermal aggregation assays identified the FI, LT, and ER peptides as interactive sequences in αB crystallin that were important for the dynamic assembly of microtubules.

**Figure 4:**
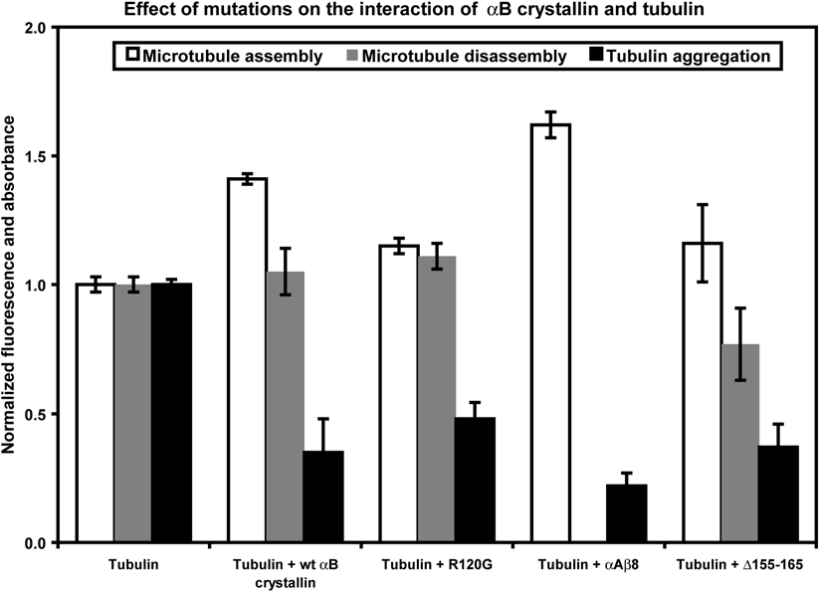
Effect of mutations in three αB crystallin interactive domains on microtubule assembly, disassembly, and tubulin aggregation. The DAPI fluorescence of assembled microtubules, disassembled tubulin, and tubulin aggregates in the absence of αB crystallin mutants was normalized to 1.0. In the presence of wt αB crystallin, microtubule assembly increased, microtubule disassembly was unchanged, and tubulin aggregation decreased. In the presence of the R120G mutant, which contains a mutation of the Arg-120 residue in the _113_FISREFHR_120_ interactive sequence of αB crystallin, microtubule assembly decreased and microtubule disassembly and tubulin aggregation were similar to wt αB crystallin. In the presences of the αAβ8 mutant, in which the β8 sequence _131_LTITSSLS_138_ of αB crystallin was replaced with the β8 sequence of αA crystallin _127_SALSCLSS_134_, microtubule assembly increased, microtubule disassembly decreased, and tubulin aggregation was unchanged relative to wt αB crystallin. In the presence of the C-terminal deletion mutant Δ155–165, microtubule assembly and disassembly were lower and tubulin aggregation was unchanged relative to wt αB crystallin. Mutagenesis of sequences in αB crystallin corresponding to the αB crystallin peptides that altered tubulin-microtubule dynamics confirmed the effects of the αB crystallin peptides on microtubule assembly/disassembly and tubulin aggregation.

Microtubule assembly and disassembly, and tubulin aggregation assays were conducted with αB crystallin mutants R120G, αAβ8, and Δ155–165, which contained mutations at sites corresponding to the FI, LT, and ER peptides respectively to confirm the results obtained with the synthetic peptides (Figure 4). Wt αB crystallin increased microtubule assembly by ∼41%, had no effect on the microtubule disassembly, and decreased the thermal aggregation of tubulin by 65%. With the αB crystallin mutant R120G, which contains a single point mutation in the _113_FISREFH**R**_120_ sequence, microtubule assembly and disassembly were unchanged while tubulin aggregation decreased. The αB crystallin mutant αAβ8, which contains multiple mutations at residues corresponding to the _131_LTITSSLS_138_ sequence increased microtubule assembly, completely inhibited microtubule disassembly, and decreased tubulin aggregation. The Δ155–165 mutant, which lacks residues 155–165 corresponding to the ER peptide, increased microtubule assembly, and decreased both microtubule disassembly and tubulin aggregation. The results confirmed the importance of the αB crystallin sequences _113_FISREFHR_120_, _131_LTITSSLSSDGV_142_, and _156_ERTIPITRE_164_ in microtubule assembly, disassembly and aggregation.

**Figure 5:**
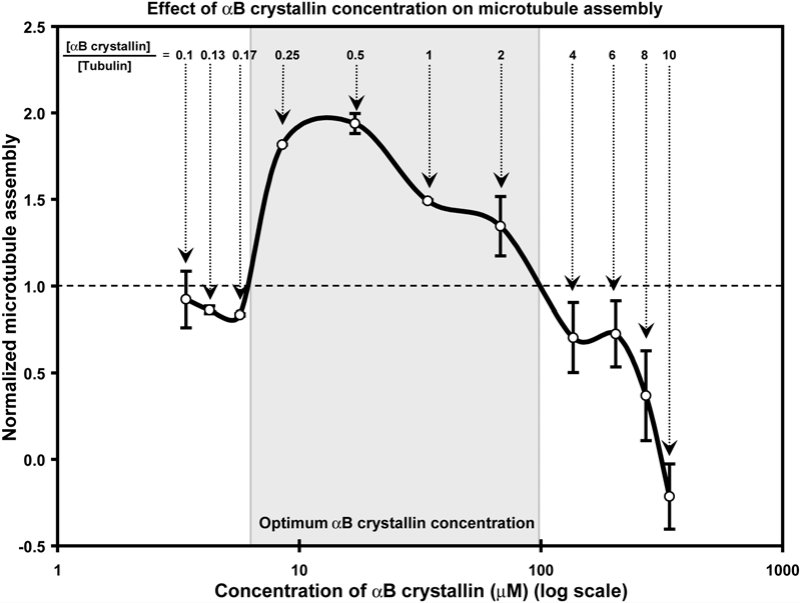
Effect of αB crystallin concentration on microtubule assembly. Microtubule assembly (Y-axis) was sensitive to the concentration of wt αB crystallin (X-axis). Microtubule assembly in the absence of αB crystallin was normalized to 1.0. The ratio of αB crystallin to tubulin for each concentration of αB crystallin is listed at the top of the plot. For αB crystallin to tubulin ratios <1∶4, microtubule assembly was unchanged at 1.0. For ratios between 1∶4 and 2∶1, microtubule assembly was >1.0 with maximum assembly observed at a tubulin to αB crystallin ratio of approximately 1∶2. For ratios >4∶1, microtubule assembly was <1.0. For a αB crystallin to tubulin ratio of 10∶1, microtubule assembly was undetectable. The variation of microtubule assembly with increasing concentrations of human αB crystallin is explained by the dynamic subunit model for the chaperone activity of αB crystallin.

To evaluate the concentration dependence of αB crystallin on the assembly and disassembly of microtubules, a fixed amount (34 µM) of tubulin was incubated with increasing concentrations of wt αB crystallin (Figure 5). At low concentrations of wt αB crystallin, no measurable effect on microtubule assembly was observed. With increasing concentrations of αB crystallin, microtubule assembly increased to a maximum and then declined at high concentrations of αB crystallin where microtubule assembly was inhibited. With respect to the ratio of αB crystallin to tubulin, the effect on assembly of microtubules was minimal when the ratio of αB crystallin to tubulin was <1∶4. When the ratio of αB crystallin to tubulin was between 1∶4 and 2∶1, the amount of microtubules formed was 35–94% higher than tubulin alone. Microtubule assembly was optimal when the ratio of αB crystallin to tubulin was approximately 1∶2. When the ratio of tubulin to αB crystallin was >2∶1 the amount of microtubules formed decreased as much as 30–63% compared to tubulin alone and no microtubules were formed when the ratio of tubulin to αB crystallin was 1∶10. Wt αB crystallin stabilized microtubules in a concentration dependent manner and was most effective within a narrow concentration range.

## Discussion

Five interactive sequences in the sHSP and molecular chaperone, human αB crystallin participate in the stabilization of tubulin/microtubules. Individual synthetic αB crystallin peptides and full-length αB crystallin mutants either promoted or inhibited microtubule assembly and disassembly suggesting a complex mechanism for the effect of wild type αB crystallin on tubulin/microtubules. Synthetic peptides corresponding to the αB crystallin sequences _131_LTITSSLSSDGV_142_ and _155_ERTIPITRE_165_ promoted microtubule assembly. In contrast, the synthetic peptide corresponding to the _113_FISREFHR_120_ sequence inhibited microtubule assembly. The remaining αB crystallin sequences _41_STSLSPFYLRPPSFLRAP_58_ and _73_DRFSVNLDVKHFS_85_ had little or no effect on microtubule assembly. The results were consistent with previous reports in which full-length wt αB crystallin interacted with tubulin and modulated the assembly of tubulin into microtubules [17], [21]. In thermal aggregation assays, the interactive sequences _113_FISREFHR_120_ and _131_LTITSSLSSDGV_142_ protected disassembled tubulin from unfolding and aggregation which was consistent with previous reports that full-length wt αB crystallin protected tubulin from unfolding and aggregation under stress [18]–[20], [22]. _113_FISREFHR_120_ and _155_ERTIPITRE_165_ are flexible and unstructured sequences in the loop region and the C-terminal extension respectively, and the _131_LTITSSLSSDGV_142_ sequence is in β strands 8 and 9 on the surface of the conserved α crystallin core domain in the αB crystallin homology model. The action of synthetic αB crystallin peptides on the assembly and aggregation of tubulin/microtubules suggests that the interaction between sHSPs and tubulin/microtubules is due to surface exposed residues that did not require specific 3D conformations and mutagenesis of these exposed residues in wt αB crystallin resulted in altered activity. In wt αB crystallin, the 3D organization of the interactive sequences may be necessary for coordinating their collective activity in response to cell stress and in control of microtubule assembly during cell proliferation.

Previous studies involving protein pin array assays, site-directed mutagenesis, and size exclusion chromatography characterized the N-terminal sequence _41_STSLSPFYLRPPSFLRAP_58_, the α crystallin core domain sequences, _73_DRFSVNLDVKHFS_85_, _113_FISREFHR_120_, and _131_LTITSSLSSDGV_142_, and the C-terminal sequence, _156_ERTIPITRE_164_ as important sequences for subunit-subunit interactions, chaperone activity, and filament interactions [26], [27], [29], [31](Figure 2). Site-directed mutagenesis of human αB crystallin demonstrated that chaperone activity was independent of complex size and that chaperone activity required exposure of the same interactive domains on the surface of αB crystallin that were used in assembly [26], [27], [29], [31], [37]. This observation is consistent with the dynamic subunit model for αB crystallin function in cells in which the dissociation of αB crystallin subunits from α crystallin complexes and/or filament networks regulates association with unfolded substrate proteins, and re-association into α crystallin-substrate complexes [37]. The relative affinity of αB crystallin for itself and selected substrate proteins explains the functional significance of the dynamic subunit model for sHSP assembly in regulation of sHSP structure and function [37].

**Figure 6:**
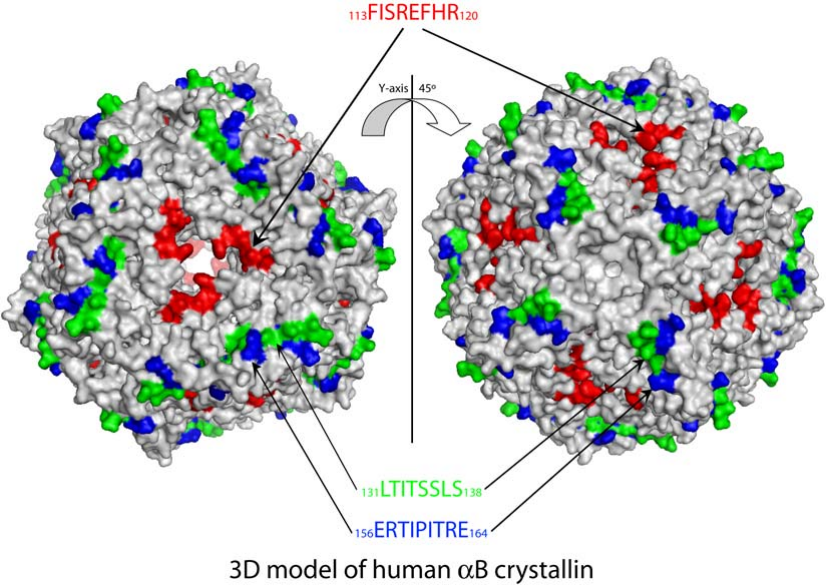
Model of the tubulin interactive sequences in the human αB crystallin complex and their importance in the assembly of microtubules. In the model, twenty-four subunits (grey) of αB crystallin form a complex which is a hollow sphere containing eight windows entering the central cavity [34], [36], [38], [61]. The αB crystallin sequences _113_FISREFHR_120_, _131_LTITSSLS_138_, and _156_ERTIPITRE_164_ that modulate tubulin-microtubule dynamics are in red, green, and blue respectively. The _113_FISREFHR_120_ sequence, which inhibits microtubule assembly is exposed on the surface of the hollow αB crystallin complex. _113_FISREFHR_120_ sequences from three separate αB crystallin subunits surround each of the eight windows that lead into the hollow core of the complex. In contrast, the _131_LTITSSLS_138_ and _156_ERTIPITRE_164_ sequences, which promote microtubule assembly, are sites of subunit-subunit interactions in αB crystallin with limited exposure on the surface of the complex. For these sequences to interact with tubulin and promote microtubule assembly, dissociation of the subunits from the complex is required. In contrast, tubulin binding to the inhibitory _113_FISREFHR_120_ sequences can occur on the surface of the complex. The computed model for the human αB crystallin complex was based on the *Methanococcus jannaschii* sHSP16.5 twenty-four subunit crystal structure described previously [62].

The observation that the same αB crystallin domains interact with unfolding substrate proteins during chaperone activity and interact with tubulin during microtubule assembly is consistent with the dynamic subunit model for sHSP function. The structural importance of the LT and ER sequences in the normal dynamic assembly and disassembly of αB crystallin complexes and the functional role of the LT and ER sequences in promoting microtubule assembly further supports the dynamic subunit exchange model for sHSP function [26], [34], [36]–[41] (Figures 2 and 6). At high αB crystallin concentrations (>100 µM) and large αB crystallin∶tubulin ratios (>4∶1), where it is expected that αB crystallin was predominantly assembled into complexes, the LT and ER sequences in apposed αB crystallin subunits interacted with each other and were unable to promote microtubules assembly (Figure 6). In contrast, the FI sequence, which inhibited microtubule assembly, remained accessible on the surface of the complex for interactions with tubulin (Figure 6). At low αB crystallin concentrations (<8 µM) and small αB crystallin∶tubulin ratios (<1∶4), the amount of αB crystallin present was insufficient to modulate microtubule assembly and there was little or no effect on normal microtubule assembly. At intermediate αB crystallin concentrations (8–100 µM) and αB crystallin∶tubulin ratios between 1∶4 and 2∶1, the LT and ER sequences were exposed on the surface of disassembled αB crystallin subunits to stabilize microtubules and promote the assembly of additional microtubules. The overlap between interactive sites for assembly, chaperone activity, and filament interactions and their 3D organization on the surface of αB crystallin subunits supports the dynamic subunit model for the physiological function of αB crystallin, which involves the dynamic association, dissociation, and re-association of αB crystallin with itself and target substrate proteins including tubulin. *In vivo*, the effect of αB crystallin on microtubule assembly is determined by the dynamics of the equilibrium between free αB crystallin subunits and αB crystallin subunits self associated in oligomers or assembled in complexes with other protein substrates. If this interpretation is correct, measurement of the relative affinities between αB crystallin subunits and selected substrates under normal and stress conditions will confirm the hypothesis that dynamic subunit assembly is responsible for the observed relationship between microtubule assembly and αB crystallin concentration. Quantitative studies are being conducted using surface plasmon resonance (SPR) to test this hypothesis.

The results are consistent with the importance of sHSPs in the amyloid cascade pathway: formation of amyloid oligomers/fibrils→hyperphosphorylation of tau→disruption of tau-tubulin interactions→formation of neurofibrillary tangles (NFTs)→neurodegeneration [42]–[44]. Although various studies support the amyloid cascade hypothesis, the mechanism of interaction between amyloid plaques and NFTs remains uncharacterized. Although the constitutive expression of sHSPs in the normal brain is low, sHSPs including αB crystallin are major constituents of amyloid plaques in Alzheimer's disease patients [45]–[47]. A recent study reported that there is a marked increase in the expression of αB crystallin and sHSP25 in transgenic mouse models of familial amyotrophic lateral sclerosis, Parkinson's disease, dentato-rubral pallido-luysian atrophy and Huntington's disease [48]. The resulting high concentration of αB crystallin in response to the toxic stress of amyloid-β can destabilize microtubules. This hypothesis is consistent with the association of αB crystallin with extracellular neurofibrillary tangles seen in Alzheimer's disease patients [49] but not intracellular NFTs [45]. Microtubule stabilizers may have therapeutic value in neurodegenerative diseases such as Alzheimer's disease where hyper-phosphorylation of the microtubule associated protein tau results in the disintegration of microtubules and the formation of NFTs [50], [51].

Modulation of microtubule assembly is of great interest in the development of new cancer treatments [50], [52]–[56]. The identification of microtubule stabilizing peptides may have therapeutic significance in the development of novel bioactive peptides as anti-cancer agents [57], [58]. Peptides that prevent microtubule disassembly can interrupt mitosis, prevent cell division, and trigger apoptosis. The effectiveness of two of the most important anti-cancer drugs today, Paclitaxel and Docetaxel whose mechanism of action involves stabilization of microtubules to disrupt cell division is limited by undesirable side effects including drug resistance. The αB crystallin peptides LTITSSLSSDGV and ERTIPITRE that alter tubulin↔microtubule dynamics can be developed into safe new therapeutics for cancer, Alzheimer's disease, and taupathies.

In summary, interactive sequences on the surface of αB crystallin that selectively recognize and stabilize tubulin can have dual effects on microtubule assembly that depend upon the αB crystallin:tubulin ratio. Favorable ratios stabilize tubulin and promote microtubule assembly and unfavorable ratios inhibit microtubule assembly. To our knowledge, this is the first experimental evidence for the functional importance of the dynamic subunit mechanism of sHSP assembly.
